# Calmodulin acetylation: A modification to remember

**DOI:** 10.1016/j.jbc.2021.101273

**Published:** 2021-10-02

**Authors:** Chiho Sugimoto, A.J. Robison

**Affiliations:** Department of Physiology, Michigan State University, East Lansing, Michigan, USA

## Abstract

The formation of new memories appears to require alterations in the shape and strength of synapses within the hippocampus, yet our knowledge of the molecular mechanisms underlying these changes remains incomplete. Zhang and colleagues provide new understanding of memory formation by uncovering the lysine acetyltransferase SRC3 as the key driver of the novel posttranslational modification of calmodulin (CaM) acetylation, which regulates CaM's activity and subsequent activation of CaMKII. This new pathway is demonstrated to be both necessary and sufficient for CA3→CA1 synapse long-term potentiation (LTP) and fear memory formation, and this approach may act as a blueprint for future investigation of the role of acetylation of other proteins in neuronal functions.

Learning and memory formation are central cognitive functions underlying behavioral changes required for animals to adapt and thrive. For many decades, the cellular and molecular underpinnings of learning have been explored in an attempt to both understand the mind and uncover potential therapeutic inroads for diseases affecting cognition and memory. Long-term potentiation (LTP), a discreet and persistent increase in synaptic strength following chronic synaptic activity, is widely considered to be a critical cellular correlate of memory. Dozens of genes, epigenetic mechanisms, protein pathways, and posttranslational modifications have been identified as critical for the many forms of LTP that occur in different brain regions, neuronal subtypes, and even specific circuits (1). The most well-studied neuronal connection at which LTP occurs is the hippocampal CA3 to CA1 synapse, and of the hundreds of molecular pathways that are critical for plasticity at this site, the most famous is probably calcium-driven calmodulin (CaM) regulation of the Ca^2+^/CaM-dependent protein kinase 2α (CaMKIIα) (2, 3). CaMKIIα activity drives LTP by increasing the number of AMPA-type glutamate receptors trafficked to the synaptic membrane and phosphorylating them to increase conductance, thus increasing strength of these synapses (4). Although the neuroscience community has studied this pathway at this specific synapse for more than 30 years, the details of its many levels of regulation and how the structure and function of each molecular player subtly contribute to the encoding of new information remain elusive. Our lack of a complete model for this and other cellular mechanisms of learning prevents us from fully unlocking the mysteries of memory formation and its dysfunction in cases such as Alzheimer's disease or intellectual disability.

In a recent issue, Zhang and colleagues (5) took a great step forward in improving our understanding of memory formation by uncovering a new enzymatic pathway leading to a novel posttranslational modification of CaM that regulates its function in CA3→CA1 synapse LTP and in learning and memory (Fig. 1). Previous work has demonstrated that posttranslational modifications of CaM, such as phosphorylation or oxidation, can affect its function and regulation of CaMKII (6, 7), but in a companion paper (8), Zhang and colleagues presented the first evidence of CaM acetylation (Ac-CaM). They demonstrate that CaM can be acetylated on any or all of three lysine residues in response to LTP or contextual fear learning, and that this increases Thr^286^ autophosphorylation and activation of CaMKIIα. Moreover, using a novel knock-in mouse in which the *Calm1* gene is mutated such that all three lysines are converted into arginine residues that cannot be acetylated, they show that Ac-CaM is necessary for both LTP and learning. In a final, stunning display of ingenuity, the authors performed viral vector-based rescue experiments with acetyl-mimicking mutant CaM to confirm this finding (8).

**Figure 1:**
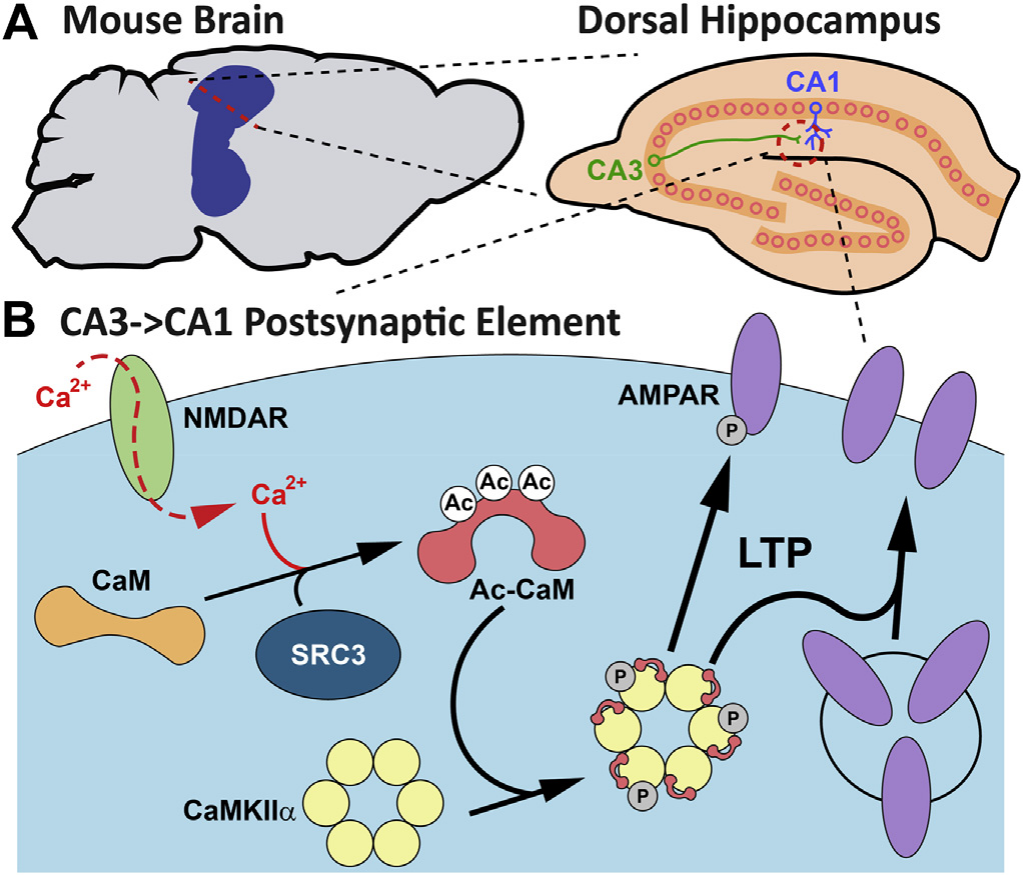
**A novel mechanism for synaptic plasticity.***A*, schematic depicting location of dorsal hippocampus in mouse brain (*left*) and a CA3→CA1 synapse within the hippocampus (*right*). *B*, within the CA1 postsynaptic element, NMDA receptors activated by glutamate allow influx of Ca^2+^, which drives the acetylation (Ac) of CaM by SRC3. Ac-CaM bound to Ca^2+^ causes CaMKIIα activation and autophosphorylation (P), driving AMPA receptors to the synapse and increasing their phosphorylation (P), key steps in long-term potentiation (LTP).

This work begged a critical question: what is the enzymatic pathway driving CaM acetylation, and is it also important in learning and LTP? In the highlighted work (5), Zhang and colleagues used a cell-based screen of lysine acetyltransferases (KATs) to identify steroid receptor coactivator 3 (SRC3) as the most active KAT for CaM. SRC3 acetylates CaM in a Ca^2+^- and NMDA receptor-dependent manner, and pharmacological and genetic inhibition of SRC3 impaired Ac-CaM and CaMKIIα activation following LTP or fear conditioning (Fig. 1). Again making use of rescue experiments with acetyl-mimicking mutant CaM, Zhang and colleagues demonstrated both the necessity and sufficiency of SRC3 acetylation of CaM for these critical aspects of learning, firmly establishing this novel pathway as a key player in hippocampal memory formation.

These findings are exciting and novel as they are the first to demonstrate a role for SRC3 in neuronal functioning and learning. SRC3 is a member of the p160 steroid receptor transcriptional coactivator family and has been implicated as an oncogene in breast cancer. As SRC3 inhibition is being targeted as a therapeutic inroad for treatment of triple-negative breast cancer (9), the current work will be critical for evaluation of any cognitive side effects of such potential chemotherapies in the future. In addition to the direct implications of this work for our understanding of learning and treatment of disease, the discovery and validation approach in these studies can act as a blueprint for investigating acetylation of other proteins in other important neuronal functions, behaviors, and potential disease treatments.

As with any exciting research project that drives the field forward, there are limitations to the current work that need to be addressed in follow-up studies. For instance, the authors go to great length to uncover the stoichiometry of CaM acetylation *in vivo*, and they find that about 93% of CaM remains unmodified, a surprisingly high proportion. Since the knock-in mouse and shRNA experiments so clearly demonstrate the functional necessity of Ac-CaM, future lines of study will need to determine whether subcellular pools of Ac-CaM exist that drive the functional consequences of acetylation. Since LTP can be induced by compartmentalized increases of Ca2+ concentrations in postsynaptic dendritic spines, localized SRC3 activation may produce locally higher levels of Ac-CaM that specifically affect the strength of those synapses. Subcellular fractionation and/or confocal microscopy may prove vital to address this question. Moreover, there are three genes encoding essentially identical CaM proteins in mice, and all are expressed in the hippocampus, making it unclear why a knock-in mutation at the *Calm1* gene alone could have such dramatic effects, and why CaM generated from *Calm2* or *Calm3* cannot compensate in the acetylation pathway. Creating additional mutant mice to address this question may be overkill, but we and others have developed viral vectors allowing direct modification of genomic DNA in mouse hippocampal neurons (10), which may facilitate easier interrogation of *Calm2* and *Calm3* contributions to Ac-CaM function. In addition, although the current studies demonstrate that acetylation of CaM is a Ca^2+^-dependent process, the order of operations and potential interactive kinetics of Ca^2+^ binding, acetylation, and binding/activation of CaMKII by CaM is yet to be determined. Finally, the fear conditioning used in these studies is not truly specific to hippocampal function, as fear learning can be driven by the amygdala. Future studies using the Ac-CaM-deficient knock-in mouse assessing performance in water maze or other spatial learning tasks will be crucial.

Nonetheless, the series of experiments conducted by Zhang and colleagues provide new insight into the molecular mechanisms of synaptic plasticity and learning and open up an exciting new avenue for study of Ca^2+^-dependent regulation of neuronal function and learning.

## Conflict of interest

The author declares that they have no conflicts of interest with the contents of this article.
